# A Better Disinfectant for Low-Resourced Hospitals? A Multi-Period Cluster Randomised Trial Comparing Hypochlorous Acid with Sodium Hypochlorite in Nigerian Hospitals: The EWASH Trial

**DOI:** 10.3390/microorganisms10050910

**Published:** 2022-04-26

**Authors:** Giorgia Gon, Lucia Dansero, Alexander M. Aiken, Christian Bottomley, Stephanie J. Dancer, Wendy J. Graham, Olivia C. Ike, Michelle Lewis, Nick Meakin, Obiora Okafor, Nkolika S. Uwaezuoke, Tochi Joy Okwor

**Affiliations:** 1 Department of Infectious Disease Epidemiology, London School of Hygiene and Tropical Medicine, London WC1E 7HT, UK; alexander.aiken@lshtm.ac.uk (A.M.A.); christian.bottomley@lshtm.ac.uk (C.B.); wendy.graham@lshtm.ac.uk (W.J.G.); 2 Department of Clinical and Biological Sciences, University of Turin, 10124 Turin, Italy; lucia.dansero@unito.it; 3 Department of Microbiology, NHS Lanarkshire, Airdrie ML6 0JS, UK; stephanie.dancer@lanarkshire.scot.nhs.uk; 4 School of Applied Science, Edinburgh Napier University, Edinburgh EH10 5DT, UK; 5 Nigeria Centre for Disease Control, Abuja 240102, Nigeria; olivchina@gmail.com (O.C.I.); obiora.okafor@ncdc.gov.ng (O.O.); tochi.okwor@ncdc.gov.ng (T.J.O.); 6 Aqualution Systems Limited, Duns TD11 3HS, UK; michelle.lewis@aqualution.co.uk (M.L.); nick.meakin@aqualution.co.uk (N.M.); 7 Federal Medical Centre, Abuja 900211, Nigeria; nscuwaezuoke@yahoo.com

**Keywords:** hypochlorous acid, disinfectant, hospital, environmental hygiene, Nigeria

## Abstract

Environmental hygiene in hospitals is a major challenge worldwide. Low-resourced hospitals in African countries continue to rely on sodium hypochlorite (NaOCl) as major disinfectant. However, NaOCl has several limitations such as the need for daily dilution, irritation, and corrosion. Hypochlorous acid (HOCl) is an innovative surface disinfectant produced by saline electrolysis with a much higher safety profile. We assessed non-inferiority of HOCl against standard NaOCl for surface disinfection in two hospitals in Abuja, Nigeria using a double-blind multi-period randomised cross-over study. Microbiological cleanliness [Aerobic Colony Counts (ACC)] was measured using dipslides. We aggregated data at the cluster-period level and fitted a linear regression. Microbiological cleanliness was high for both disinfectant (84.8% HOCl; 87.3% NaOCl). No evidence of a significant difference between the two products was found (RD = 2%, 90%CI: −5.1%–+0.4%; *p*-value = 0.163). We cannot rule out the possibility of HOCl being inferior by up to 5.1 percentage points and hence we did not strictly meet the non-inferiority margin we set ourselves. However, even a maximum difference of 5.1% in favour of sodium hypochlorite would not suggest there is a clinically relevant difference between the two products. We demonstrated that HOCl and NaOCl have a similar efficacy in achieving microbiological cleanliness, with HOCl acting at a lower concentration. With a better safety profile, and potential applicability across many healthcare uses, HOCl provides an attractive and potentially cost-efficient alternative to sodium hypochlorite in low resource settings.

## 1. Introduction

Low-resourced hospitals in African countries and elsewhere face substantial challenges in ensuring environmental hygiene standards are maintained, as shown in both the West African Ebola virus epidemic and the COVID-19 pandemic [1]. The importance of environmental hygiene in Africa is heightened by role of antimicrobial resistance (AMR), reported to have the highest rates of death in sub-Saharan Africa [2].

One substantial problem for these hospitals is a continued reliance on commercial sodium hypochlorite (NaOCl)—commonly called “bleach”. This product requires accurate daily dilution with water prior to use, but most African hospitals, as with other hospitals in other low resource settings, do not provide any formal training for cleaning staff on use of disinfectants [3]. Additionally, supply chains for hypochlorite products can be problematic with lack of quality control, no regard for expiry dates, and inappropriate storage conditions. Indeed, bleach products available in Africa are often of inconsistent quality, affecting potency [4]. Other limitations of bleach include irritation of skin and mucous membranes if used in poorly ventilated areas, ecological toxicity of key ingredients and by-products, corrosion of surfaces cleaned (including many metals, rubber and some plastics) and bleaching of fabrics.

An ideal disinfectant product would achieve a high kill rate of potential pathogens whilst maintaining a good safety profile with low toxicity while permitting simple in-use quality control. These product characteristics are especially important when the environmental cleaning burden is high and there is often overcrowding with insufficient isolation facilities to allow segregation of high-risk infectious patients. This is the scenario faced by many hospitals in low resource countries.

The well-known limitations of sodium hypochlorite have encouraged interest in alternative disinfectants for use in hospitals, and in particular the use of locally-sourced disinfectants to avoid issues with product degradation. One alternative disinfectant can be produced locally by saline electrolysis [5,6]; hence, these products may be termed electrolysed water. The active ingredients resulting from electrolysis include chlorine, hypochlorous acid and hypochlorite ions or a combination of these. Of these, hypochlorous acid offers the most useful antimicrobial properties—the pure form of this compound can be highly microbiocidal with minimal toxicity. This type of electrolysis can achieve a higher purity of available hypochlorous acid than can be delivered using traditional bleach manufacturing process [7]. Sodium hypochlorite solutions (ie bleach) also largely achieve microbicidal effects through conversion of hypochlorite to hypochlorous acid, but with many inadvertent by-products. Modern electrolysis cells can produce hypochlorous acid in a neutral solution with a pH of approximately 6–8 [8,9].

Aqueous hypochlorous acid is emerging as a potent and environmentally safe disinfectant available. This compound, in appropriate concentrations, can rapidly inhibit or kill a wide range of human pathogens [6], including bacteria and spores, viruses such as the SARS-CoV-2 coronavirus [10,11], fungi, protozoa and mycobacteria [6,12].

Correctly managed, electrolysis can provide a relatively pure solution of hypochlorous acid along with other active oxidants and free radicals [7]. Improvements in the manufacturing process for hypochlorous acid (HOCl) offers stable solutions of HOCl in industrial quantities and is widely used in infection control in high-income countries and the food sector in low and middle income countries. Specifically, Salvesan™ is a disinfectant produced by Aqualution (Duns, UK), which is generated through electrolysis. Its chemical composition is 99% HOCl and 1% hypochlorite. This product can achieve rapid surface disinfection at a lower concentration compared with standard commercial sodium hypochlorite and hence has a higher safety profile. The pH of this product is ~7 which permits stability over a 12-month shelf life. This contrasts with other commercial hypochlorous acid products, which are only usually viable for hours to days [13]. The ecological residue from HOCI is negligible because it reverts to salt and water, and is therefore less toxic for both environment and users than bleach (sodium hypochlorite) whose degradation products include sodium chlorate and trihalomethanes which are substances of concern, being recognized carcinogens.

Previous studies have demonstrated that hypochlorous acid produced by electrolysis and traditional sodium hypochlorite solution can have similar levels of microbiocidal effectiveness [7,8,13] but we are not aware of any previous randomised trials of effectiveness in a low resourced settings. We aimed to assess the efficacy of HOCl against standard sodium hypochlorite (both used at recommended concentrations) for hospital cleaning across six wards of two hospitals in Abuja, Nigeria, using a cross-over randomised design called the EWASH Trial.

## 2. Materials and Methods

### 2.1. Study Design

The study was a non-inferiority double-blind cluster-randomised controlled trial with multiple period crossover random allocation. Clusters were randomly allocated to one of two crossover sequences between the control and intervention states. The clusters were the female unit (surgical and medical wards), the male unit (surgical and medical wards), and maternity (antenatal and postnatal wards).

### 2.2. Study Population

The two study hospitals are public-sector hospitals in Abuja Federal Capital Territory (FCT), Nigeria. These are one secondary (district) and one tertiary hospital. Three clusters were selected in each of the two hospitals (female and male units, and maternity); each cluster was composed of two wards—these were usually adjacent and shared the same cleaning staff. There was no crossing over or sharing of cleaners and cleaning materials between study clusters.

**Figure 1:**
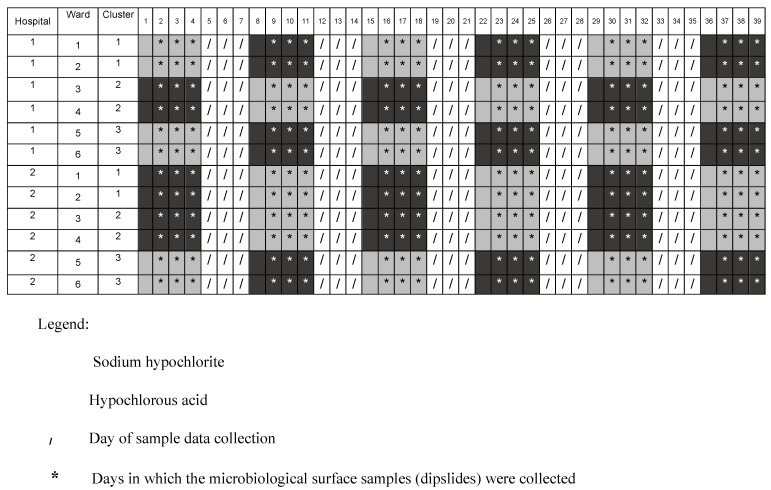
Intervention structure over a six-week period.

We sampled 28 surfaces from each of five cluster; in the sixth cluster we had 26 surfaces. The same surfaces were sampled across the study period (see Figure 1 for cohort structure). The surfaces were selected within patient zones (bed and adjacent items dedicated to the patient) and included those most frequently touched by health workers and patients: specifically, the mattress, bedframe, bedside-locker, table, and chair [14,15,16].

### 2.3. Intervention

The intervention was the application of hypochlorous acid for surface disinfection (trade name Salvesan, Aqualution^®^). The product was manufactured in February 2021 in the UK and arrived in Abuja in May 2021. The intervention lasted six weeks, from the 21 June 2021 until the 1 August 2021.

The intervention product has received the approvals for the decontamination of hospital surfaces through the relevant institutional EU bodies (BS/EN 1276, BS/EN 13727, BS/EN 1500, BS/EN 13704, BS/EN 1650, BS/EN13697). To our knowledge, this product has never previously been used in Nigeria for hospital cleaning purposes.

Sodium hypochlorite was used as control product; this is the product usually supplied to the hospital and recommended by the local government. This was purchased from Hypo Hygiene Products Limited (https://hypo.com.ng/ accessed on 20 May 2021) the week prior to the study and stored appropriately throughout. Hypochlorous acid had a dilution of 0.015% (=150 ppm), whereas sodium hypochlorite was diluted to 0.05% (=500 ppm). The project manager prepared both products each morning and delivered them to the assigned wards. For hypochlorous acid, this was just transfer of the product from the storage containers to the study spray bottles, since the product was already at the correct dilution. For sodium hypochlorite, dilution of the product with water (from 3.5% to 0.05%) was required before decanting it into study spray bottles. Both products are colourless, clear liquids and emanate a chlorine-like odour, with that from sodium hypochlorite a little stronger. The disinfection process was standardised to include the number of sprays for a defined surface area and site of application. Kitchen roll (Rose) was applied for the application of both products. The study utilised a cross-over schedule with 4 days of intervention followed by 3 days of “wash-out” period to avoid cross-contamination between products. Intervention and wash-out periods were repeated six times with crossover of intervention and control between the two cluster sequences.

During the wash out period, hospital staff reverted to the standard disinfectant, namely sodium hypochlorite. However, during these wash-out periods the project manager did not provide the product to the wards—preparation was managed by the hospital staff according to routine practice.

All hospital cleaners participated in a short training workshop (half a day) focused on surface disinfection technique based on the TEACH CLEAN package (link: https://www.lshtm.ac.uk/research/centres/march-centre/soapbox-collaborative/teach-clean; accessed on 1 May 2021) and the visual cleaning guidelines were available in each ward. This is the only CDC-recommended available training for LMICs (link: https://www.cdc.gov/hai/pdfs/resource-limited/environmental-cleaning-RLS-H.pdf, accessed on 1 of May 2021). This was done to ensure all cleaners had a similar baseline level of training.

### 2.4. Outcome and Data Collection

The outcome was measured daily during three days of intervention for each week (from day 2 to day 4 for each period; see Figure 1).

Microbiological cleanliness was measured using double-sided dipslides (Dimanco Ltd., Henlow, UK), which is a widely-used quantitative method for measuring surface microbiological cleanliness in hospitals and elsewhere [17]. The dipslides were coated with nutrient agar on one side to capture total Aerobic Colony Counts (ACC/cm^2^) and a selective agar (Baird-Parker media) on the reverse to aid detection of *Staphylococcus aureus*. Both sides were applied consecutively at uniform pressure to adjacent areas of the sampling site without overlap.

After sampling each day, dipslides were transported to the laboratory within two hours and incubated on the day of sampling under aerobic conditions for 24 h at 37 °C. After incubation, colonies were enumerated by visual inspection and categorized as: “No growth” (0 cfu/cm^2^), “Scanty growth” (>0 to 2.5 cfu/ cm^2^), “Light growth” (≥2.5 to 12 cfu/cm^2^), “Moderate growth” (≥12 to 40 cfu/cm^2^), “Heavy growth” (≥40 cfu/cm^2^), and “Confluent growth” (overlapping colonies making precise enumeration impossible). Any potential *S. aureus* colonies with appropriate colony morphology were tested for presence of coagulase using the slide latex agglutination. Coagulase-positive colonies were subcultured onto blood agar and re-incubated for a further 24 h at 37 °C in air, before repeating coagulase testing. Sample that were repeated positives were subcultured onto a selective media (CHROMagar™ *Staph aureus*) for species confirmation.

For quality control purposes, every 10th dipslide was quantitatively checked by an internal 2nd reader and every 20th dipslide by an external 3rd reader.

#### 2.4.1. Primary Outcome

Aerobic Colony Counts (ACC)/cm² determined the primary outcome of the study. The outcome was binary with surface cleanliness defined as positive (pass) if <2.5 cfu/cm^2^ and negative (fail) if ≥2.5 cfu/cm^2^. This is a standardised method and cut-off to use for measuring microbiological cleanliness of hard surfaces in hospital settings [18].

#### 2.4.2. Secondary Outcome

Presence of *S. aureus* was the secondary outcome of the study [18]. This outcome was also binary with surface cleanliness defined as positive (absence of *S. aureus*) or negative (*S. aureus* confirmed). *S. aureus* an important human pathogen and a useful indicator of surface cleanliness [19].

### 2.5. Randomisation and Blinding

A computerised system was used to randomly allocate the clusters to one of the two intervention-control sequences. The study design permitted double-blinded methodology so that all participants (hospital staff, data collectors and laboratory staff) were blinded to the product they were using. We used identical bottles for delivering both products. The bottled were provided daily with the intended product by the project manager to each ward, with each bottle allocated a serial number to allow tracking. In addition, the lab personnel were blinded to the exact outcome measure of the study; they categorized the study outcomes based on the six categories previously explained and not the primary binary indicator which was used in the analysis.

### 2.6. Sample Size

The study design was expected to have 86% power to demonstrate non-inferiority at a 5% margin. This prediction assumed the absence of within-ward and within-individual correlation and was therefore conservative because both types of correlation are expected to increase power in a cross-over design [20]. These calculations also assumed: (1) 50% prevalence of the cleanliness outcome and (2) that non-inferiority would be assessed using the lower bound from a 95% 1-sided confidence interval (note that this is the same as the lower bound of 90% 2-sided interval).

### 2.7. Quality Control of HOCl

Each week a sample of HOCl was tested to check temperature and pH. The expected pH range was 6.5–7.5. The product was kept within the 5–35 °C temperature range as recommended by the manufacturer. Direct contact with sunlight and organic substances could deactivate the product, hence it was kept in a dark and clean environment.

### 2.8. Focus Group Discussions

A focus group discussion (FGD) was conducted in each hospital at the end of the trial to understand the participants’ perception of the two products, as well as the barriers and the enablers encountered during the trial for product application. The facilitator made available the two products during the discussion to the FGD participants to enquire about them during the FGD. Seven participants were included in one hospital and four in the other. Participants included cleaners and supervisors.

### 2.9. Analysis

Data were stored in RedCAP and analysed with Stata 16 software. We began by conducting a descriptive analysis in which we compared the proportion of surfaces meeting the cleanliness standard (ACC < 2.5 cfu/cm^2^) between control and intervention samples.

Then, accounting for the cross-over design, we aggregated the data at the cluster-period level and fitted a linear regression model. In addition to a binary indicator for treatment, the regression included week and cluster as fixed effects. We used the estimated treatment effect and associated confidence interval obtained from this regression model to assess non-inferiority. Our criterion for non-inferiority was that the lower bound of the 1-sided 95% confidence interval of the difference in cleanliness (treatment minus control) should not exceed −5%. In practice we evaluated this criterion via a 2-sided 90% confidence interval since both intervals share the same lower bound. Missing data were not included in the model.

### 2.10. Ethics

The study received ethics approval from LSHTM and Nigeria’s National Health Research Ethics Committee approval as well as permission from the participating hospitals (Federal Medical Centre Abuja’s HREC (Health Research Ethics Committee) and Federal Capital Territory Administration (FCTA) Health and Human Services Ethics Committee). During the pilot phase, we collected written consent from hospital managers and all cleaners that participated in the study. Written consent was also gathered before the FGDs. Cleaners were told that no personal information would be collected or stored.

### 2.11. Data Sharing

The data is available in the Supplementary Material—Table S1. We did not include hospital names or hospital identifiers to avoid potential identification of these.

## 3. Results

A total of 2983 surface samples were collected over a six-week period (from the 21 June 2021 until the 1 August 2021), of which 1495 were from surfaces disinfected with sodium hypochlorite and 1488 from those disinfected with hypochlorous acid.

Table 1 reports the percentages of cleanliness by treatment received. Overall, the percentage of clean samples (<2.5 CFU/cm^2^) was high for both cleaning strategies with 84.8% for hypochlorous acid and 87.3% for sodium hypochlorite (see Table 1). With regards to sub-categories of ACC levels, the two products performed similarly. There were very few missing samples (~1%), most of which occurred in week 1 (data not shown). These were either missed surfaces or error codes. There were a very small number of dipslides where the enumeration was problematic (“Confluent” category) which might suggest excessive incubation.

Overall, the number of samples collected was slightly smaller than anticipated and there was a small amount of imbalance between the clusters (see Table 2).

Overall, the two products performed similarly in each of the clusters (Table 2) with cluster range of 79.2–92.1% for hypochlorous acid and 80.8–93.2% for sodium hypochlorite.

**Figure 2:**
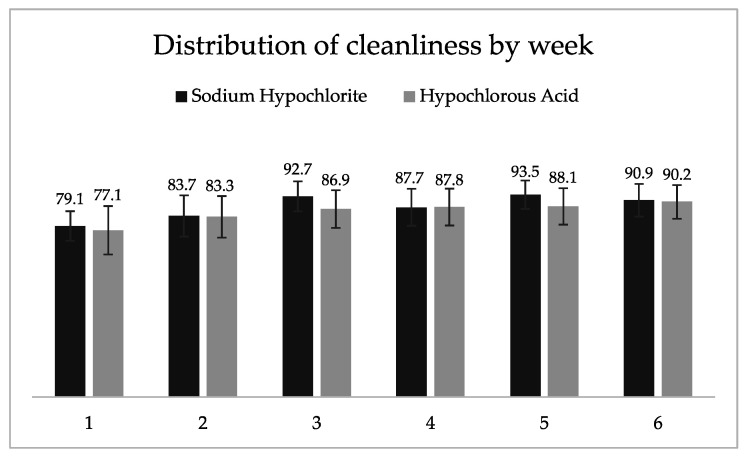
Percentage of microbiological cleanliness by treatment and by week.

As shown in Figure 2, the two products performed similarly during each of the 6 weeks of the study.

Overall, the difference between the products was 2%, indicating a slightly higher dipslide pass rate for sodium hypochlorite (see Table 3). With these data, we are confident that there was no evidence of a difference between the two cleaning fluids (90%CI: −5.1%–+0.4%), *p*-value = 0.163; however, we did not meet the non-inferiority we set ourselves and hence we cannot rule out the possibility of HOCl being inferior by up to 5.1 percentage points.

### 3.1. Isolation of S. aureus

Overall, only a small samples (24 dipslides for sodium hypochlorite and 19 for hypochlorous acid) underwent further testing for the presence of *S. aureus*. The number of *S. aureus* isolates identified was low in both treatment arms, with 4 (0.3%) when sodium hypochlorite was applied and 5 (0.3%) when HOCl was applied. The small number of failure events in this secondary outcome did not allow further statistical analysis.

### 3.2. Quality Control

The pH of hypochlorous acid was measured weekly from random bottles and ranged from 6.55 to 7.1, therefore within the necessary pH range for product stability as recommended by the manufacturer.

### 3.3. FGD Results

None of the participants reported any difference in using the products and none realised that the products were switched weekly. Positive feedbacks were given for the overall efficacy of both products in term of cleanliness and usage, however, the participants expressed some concern regarding the usage of kitchen roll, suggesting a different absorbent material for the application of the product.

Participants reported that more information could be given to the patients about the cleaning fluids as the black spray bottles made some patients feel uncomfortable as they did not know its content.

## 4. Discussion

Using a multi-period crossover design across two Nigerian hospitals, we demonstrated that HOCl and sodium hypochlorite have a similar efficacy based on overall microbiological outcome, with HOCl acting at a lower concentration. We could not measure the efficacy of the two products on the secondary outcome of *S. aureus* because surface samples yielded small number of isolates for this pathogen. With a better safety profile, no environmental residue, and potential applicability across many healthcare cleaning requirements, HOCl therefore provides an attractive alternative to sodium hypochlorite for low-income hospitals.

We found no evidence of a difference between the two products (90%CI: −5.1%–+0.4%, *p*-value = 0.163) in terms of surface microbiological cleanliness. The two products were, on average, only 2% difference indicating a slightly higher dipslide pass rate for sodium hypochlorite (see Table 3). We cannot rule out the possibility of HOCl being inferior by up to 5.1 percentage points and hence we did not strictly meet the non-inferiority margin we set ourselves; however, even a maximum difference of 5.1% in favour of sodium hypochlorite would not suggest there is a clinically relevant difference between the two products. Overall, there was a small amount of imbalance between the clusters which slightly affected our power.

We successfully blinded the products for all hospital staff including those who clean. The cleaners did not realise that the products were switched weekly, as did laboratory staff who were analysing the outcome data. In terms of bias, because during the wash-out period the hospital staff were using sodium hypochlorite which they were preparing themselves, this may raise a concern for carry-over of the effect of NaOCl in the HOCl periods. This could have led to a bias towards the null hypothesis that products perform similarly.

Overall, hard surface cleanliness, as measured by an objective, standardized method, was high in this trial (~86%). Usually, the range expected in hospitals in low-resourced settings would be expected to be in the range of 30–50% [16,21]. Reasons for this low-level of cleanliness are numerous, including the lack of formal training for cleaning staff and limited resources for environmental hygiene [22]. Most likely, the good level of cleanliness in this study was due to a combination of the training received at the beginning of the trial and also from a Hawthorne effect from daily presence of the data collectors. This does not affect our conclusions, as the main aim of the trial was to compare the two products, rather than to measure the absolute level of cleanliness in this context.

Three characteristics make HOCl particularly suitable for low-resourced hospitals. First, it does not require further dilution with water at the point of use; this is important when there is a lack of formal training on environmental hygiene. Even where formal training is available, such as in many high-income countries, dilution of disinfectants remains an issue. Second, it has a better safety profile compared with sodium hypochlorite because it does not have toxicity or corrosive properties, either from the active ingredients or the breakdown products. This safety profile makes it particularly attractive for cleaning in the near-patient zone [9]—where cleaning matters the most—for a variety of vulnerable patients, including newborns. Improving hospital cleaning could help tackle antimicrobial resistance transmission which is rampant across Africa [2,23]. Third, it can be used for other key applications in the healthcare setting including treatment of drinking water, cleaning delicate equipment without risk of corrosion, antisepsis and wound care [12]. The latter is important because it could prevent over reliance on antibiotics.

The discussion about alternative disinfectants is timely because hundreds of hospitals in low-income countries are in the process of switching to local production of bleach to ensure a better product quality than the ones they can procure. This has accelerated in the context of the COVID-19 pandemic. So, switching to local production of a better product, that could also be used for a wider set of healthcare applications is an attractive proposition. Other considerations to why now is important include environmental implications of the product being used: locally generated HOCl could potentially be produced using solar power, and it does not damage the environment because it reverts to salt and water. Finally, a modern portable electrolysis cell unit can currently produce ~1400 litres of hypochlorous acid per day which is potentially sufficient to support multiple hospitals on several potential healthcare uses. We speculate, based on local costs identified in this research, that producing hypochlorous acid locally could be substantially cheaper (approximately half the price) than commercially-available sodium hypochlorite purchased in Abuja.

The wider aim of improving environmental hygiene in LMICs, is of course not limited to a discussion on the disinfectant properties. Adequate training, supplies and human resources are all essential for maintaining hospitals clean. Indeed, during the COVID-19 pandemic, hospitals across the world observed dramatic reductions in common forms of healthcare-associated infection thanks to an increased adherence to infection prevention practices [24].

A limitation of this study is that, although we intended to, we did not measure free chlorine in both products immediately prior to use due to limitation of the available equipment. In terms of understanding the potential role of hypochlorous acid in healthcare use in LMIC, a limitation of this study is that we used a product being shipped into Nigeria from the UK—this means we cannot formally examine the relative costs of the two products, which is potentially a major advantage of hypochlorous acid. Future studies should test the effectiveness of HOCl produced locally and hence also answer the multiple questions related to building a sustainable supply chain for hospital use. An important product characteristic of hypochlorous acid, such as Salvesan, is that it needs to be kept in opaque bottles to inhibit product degradation from UV light exposure, and it should also be kept below 35 degrees. The shelf life of this particular product is 12 months. These are important considerations for use of this product in hot climates and need to be further tested. Finally, we were also not able to test the quality of the sodium hypochlorite purchased locally; however, other comparison studies in high-income settings where disinfectants undergo greater quality control checks provide similar results when compared to Salvesan [7].

In conclusion, in randomized cross-over trial in two hospitals in Nigeria, we demonstrated that HOCl and sodium hypochlorite have a similar efficacy in achieving microbiological cleanliness, with HOCl acting at a lower concentration. With a better safety profile, no environmental residues and potential applicability across many healthcare uses, locally-generated HOCl provides an attractive and potentially cost-efficient alternative to sodium hypochlorite.

## Figures and Tables

**Table 1:** Distribution of cleanliness by treatment and by ACC category.

	Sodium Hypochlorite % (*N* = 1495)	Hypochlorous Acid % (*N* = 1488)	Total % (*N* = 2983)
**Total Clean**	87.3	84.8	86.0
*No growth* (0 CFU/cm^2^)	*20.5*	*18.1*	*19.3*
*Scanty growth* (>0 to 2.5 CFU/cm^2^)	*66.8*	*66.7*	*66.8*
**Total Not clean**	11.9	14.2	13.0
*Light growth* (≥2.5 to 12 CFU/cm^2^)	*7.1*	*9.5*	*8.3*
*Moderate growth* (≥12 to 40 CFU/cm^2^)	*3.1*	*3.5*	*3.3*
*Heavy growth* (≥40 CFU/cm^2^)	*0.8*	*0.7*	*0.8*
*Confluent growth*	*0.8*	*0.5*	*0.6*
**Missing**	0.8	1.0	1.0

**Table 2:** Distribution of cleanliness by hospital and clusters.

			Clean (<2.5 CFU/cm^2^)	Not Clean (≥2.5 CFU/cm^2^)	Missing
**Hospital 1**	Cluster 1	NaClO % (N = 257)	89.5	6.6	3.9
		HOCl % (N = 252)	92.1	7.9	0
	Cluster 2	NaClO % (N = 252)	93.2	6.75	0
		HOCl % (N = 248)	85.9	10.1	4.0
	Cluster 3	NaClO % (N = 248)	90.7	8.5	0.8
		HOCl % (N = 252)	88.1	11.9	0
**Hospital 2**	Cluster 4	NaClO % (N = 252)	83.7	16.3	0
		HOCl % (N = 250)	79.2	20.0	0.8
	Cluster 5	NaClO % (N = 252)	85.3	16.7	0
		HOCl % (N = 252)	82.5	16.3	1.2
	Cluster 6	NaClO % (N = 234)	80.8	19.2	0
		HOCl % (N = 234)	80.8	19.2	0

**Table 3:** Results from linear regression with week and cluster as fixed effects.

Exposure	% (N)	Difference	S.E.	*p*-Value	90% C.I.
NaClO (ref)	87.3	1			
HOCl	84.8	−2.3%	0.0163	0.163	(−5.1–+0.4)

## Data Availability

Data are publicly available in the Supplementary Material Section.

## Supplementary Materials

The following supporting information can be downloaded at: https://www.mdpi.com/article/10.3390/microorganisms10050910/s1, Table S1: Distribution of clean samples by cluster and week.
