# Supplementary material for: A Better Disinfectant for Low-Resourced Hospitals? A Multi-Period Cluster Randomised Trial Comparing Hypochlorous Acid with Sodium Hypochlorite in Nigerian Hospitals: The EWASH Trial
Source: Microorganisms. 2022 Apr 26;10(5):910. doi: 10.3390/microorganisms10050910 (PMC9146012; doi:10.3390/microorganisms10050910)
Supplement: Supplementary file 1 [file microorganisms-10-00910-s001.zip › microorganisms-1665100-supplementary.pdf]

## Supplementary Table S1

**Table S1.** Distribution of clean samples by cluster and week.

| Cluster | Week | Percentage | N of Clean* Samples | $N_{tot}$ |
|---|---|---|---|---|
| 1 | 1 | 0.8608 | 68 | 79 |
| 1 | 2 | 0.8810 | 74 | 84 |
| 1 | 3 | 0.9405 | 79 | 84 |
| 1 | 4 | 0.9524 | 80 | 84 |
| 1 | 5 | 0.9881 | 83 | 84 |
| 1 | 6 | 0.9286 | 78 | 84 |
| 2 | 1 | 0.8429 | 59 | 70 |
| 2 | 2 | 0.9405 | 79 | 84 |
| 2 | 3 | 0.8929 | 75 | 84 |
| 2 | 4 | 0.8929 | 75 | 84 |
| 2 | 5 | 0.9405 | 79 | 84 |
| 2 | 6 | 0.9643 | 81 | 84 |
| 3 | 1 | 0.8590 | 67 | 78 |
| 3 | 2 | 0.8929 | 75 | 84 |
| 3 | 3 | 0.8929 | 75 | 84 |
| 3 | 4 | 0.8571 | 72 | 84 |
| 3 | 5 | 0.9881 | 83 | 84 |
| 3 | 6 | 0.8929 | 75 | 84 |
| 4 | 1 | 0.7250 | 58 | 80 |
| 4 | 2 | 0.8452 | 71 | 84 |
| 4 | 3 | 0.8333 | 70 | 84 |
| 4 | 4 | 0.8452 | 71 | 84 |
| 4 | 5 | 0.8333 | 70 | 84 |
| 4 | 6 | 0.8214 | 69 | 84 |
| 5 | 1 | 0.7531 | 61 | 81 |
| 5 | 2 | 0.7262 | 61 | 84 |
| 5 | 3 | 0.8810 | 74 | 84 |
| 5 | 4 | 0.8929 | 75 | 84 |
| 5 | 5 | 0.8690 | 73 | 84 |
| 5 | 6 | 0.9405 | 79 | 84 |
| 6 | 1 | 0.6538 | 51 | 78 |
| 6 | 2 | 0.7179 | 56 | 78 |
| 6 | 3 | 0.9487 | 74 | 78 |
| 6 | 4 | 0.8205 | 64 | 78 |
| 6 | 5 | 0.8205 | 64 | 78 |
| 6 | 6 | 0.8846 | 69 | 78 |

*clean defined as (<2.5 CFU/$cm^2$)
